# Association between Elevated Iodine Intake and IQ among School Children in Portugal

**DOI:** 10.3390/nu14214493

**Published:** 2022-10-26

**Authors:** Irene P. Carvalho, Bruno Peixoto, José Carlos Caldas, Ana Costa, Sofia Silva, Bárbara Moreira, Agostinho Almeida, André Moreira-Rosário, António Guerra, Cristina Delerue-Matos, Diana Sintra, Diogo Pestana, Edgar Pinto, Francisca Castro Mendes, Inês Martins, João Costa Leite, Manuel Fontoura, Maria Luz Maia, Pedro Queirós, Roxana Moreira, Sandra Leal, Sónia Norberto, Vera Costa, Virgínia Cruz Fernandes, Elisa Keating, Luís Azevedo, Conceição Calhau

**Affiliations:** 1 Medical Psychology Unit, Department of Clinical Neurosciences and Mental Health, Faculty of Medicine, University of Porto, 4200-450 Porto, Portugal; 2 CINTESIS@RISE, Faculty of Medicine, University of Porto, 4200-450 Porto, Portugal; 3 TOXRUN—Toxicology Research Unit, University Institute of Health Sciences, CESPU, 4585-116 Gandra, Portugal; 4 Department of Sciences, University Institute of Health Sciences, CESPU, 4585-116 Gandra, Portugal; 5 Center for Health Technology and Services Research (CINTESIS), Faculty of Medicine, University of Porto, 4200-450 Porto, Portugal; 6 LAQV/REQUIMTE—Department of Chemical Sciences, Faculty of Pharmacy, University of Porto, 4050-313 Porto, Portugal; 7 Nutrition & Metabolism, Nova Medical School, Faculdade de Ciências Médicas, Universidade Nova de Lisboa, 1169-056 Lisboa, Portugal; 8 CINTESIS@RISE, Nova Medical School, Universidade Nova de Lisboa, 1169-056 Lisboa, Portugal; 9 Division of Paediatric Nutrition, Integrated Paediatric Hospital, Centro Hospitalar de São João, 4200-450 Porto, Portugal; 10 Department of Gynecology-Obstetrics and Paediatrics, Faculty of Medicine, University of Porto, 4200-450 Porto, Portugal; 11 LAQV/REQUIMTE—Instituto Superior de Engenharia, Instituto Politécnico do Porto, 4249-015 Porto, Portugal; 12 Department of Environmental Health, School of Health, Instituto Politécnico do Porto, 4200-072 Porto, Portugal; 13 Clinical and Basic Immunology Unit, Department of Pathology, Faculty of Medicine, University of Porto, 4200-319 Porto, Portugal; 14 EPIUnit—Public Health Institute, University of Porto, 4200-450 Porto, Portugal; 15 Laboratory for Translational and Integrative Research on Population Health (ITR), 4200-450 Porto, Portugal; 16 Biochemistry Unit, Department of Biomedicine, Faculty of Medicine, University of Porto, 4200-450 Porto, Portugal; 17 Department of Community Medicine, Information and Health Decision Sciences (MEDCIDS), Faculty of Medicine, University of Porto, 4200-450 Porto, Portugal

**Keywords:** excessive iodine, cognition, school ages, iodine-adequate population, representative sample

## Abstract

The goal of this work was to examine whether elevated iodine intake was associated with adverse effects on IQ among school-age children in Portugal. In a representative sample of children from the north of the country, IQ percentiles by age (assessed with Raven’s Colored Progressive Matrices) were dichotomized to <50 (“below-average” IQs) and ≥50. Morning urine iodine concentrations, corrected for creatinine, were dichotomized to <250 µg/g and ≥250 µg/g, according to the European Commission/Scientific Committee on Food’s tolerable upper level of daily iodine intake for young children. Data were examined with Chi-square tests, logistic regression, and GLM univariate analysis. The sample (*N* = 1965) was classified as generally iodine-adequate (median urinary iodine concentration = 129 µg/L; median iodine-to-creatinine ratio = 126 µg/g) according to the WHO’s criteria. A greater proportion of children in the ≥250 µg/g group had below-average IQs, compared to children with less than 250 µg/g (*p* = 0.037), despite a sizable (though non-significant) proportion of children in the less-than-250 µg/g group also presenting below-average IQs, at the bottom of the iodine distribution (<50 µg/g). The proportion of below-average IQs increased with increasingly elevated iodine concentrations (*p* = 0.047). The association remained significant after the adjustment for confounders, with the elevated iodine group showing increased odds of having below-average IQs when compared with the non-elevated iodine group (OR 1.55; 95% CI 1.11–2.17; *p* = 0.011). Consistently, the former group presented a lower mean IQ than the latter (*p* = 0.006). High iodine intake was associated with lower IQs even in a population classified as iodine-adequate. These results bear on child cognition and on initiatives involving iodine supplementation.

## 1. Introduction

Children’s neurological development is a continuous process affected by various factors including dietary components. Iodine is a key micronutrient for the production of thyroid hormones, which are crucial for healthy growth and particularly for brain and neurological development [1]. However, both the deficiency and excess of iodine can pose risks to health. Iodine deficiency can lead to hypothyroidism, goiter, and cretinism, among other problems, impairing mental function among children, adolescents, and adults [2]. Although the precise mechanisms through which iodine influences brain development remain unclear, they are thought to begin with genetic expression and affect subsequent brain structures and functions [3]. Iodine deficiency might contribute to compromised neurotransmitters, myelin, and hippocampus, through the intervention of thyroid hormones in the formation and functioning of these brain structures and processes [3]. In turn, morbidities associated with excess iodine exposure can result from failures in the normal thyroidal adaptation to the excessive iodine, for example, through possible thyroid-stimulating hormone (TSH) decrease when thyroid hormone is overproduced as a result of excessive iodine, leading to problems in thyroid function regulation, or to failures in the Wolff–Chaikoff effect [4]. As a possible result of such mechanisms, excessive iodine can also induce conditions such as hyperthyroidism and hypothyroidism, autoimmune thyroid diseases, and iodine-excess goiter [2,4]. However, its role in cognitive function is less clear.

The effectiveness of iodine supplementation in reducing the risk of severe conditions associated with iodine deficiency, such as cretinism and mental retardation, has long been established, leading to public policies, namely the introduction of iodized salt, that resulted in great increases in intelligence quotient (IQ) levels across populations [1,5]. Interventions directly targeting school-age children have shown that iodine supplementation raises children’s IQ levels, even in the presence of only mild iodine deficiencies [6]. However, research is lacking on the effects of iodine supplementation on IQ among children already presenting adequate levels of iodine and among healthy populations [5,7,8].

Excessive iodine is of concern [4,9]. Regular monitoring of populations’ iodine status has been called for, not only in view of decreasing levels but also in view of excessive and above-the-recommended levels of iodine [2]. In addition to the possible risks for physical health, excessive iodine might impact children’s cognitive function, and it might do so in less obvious ways than is the development of cretinism or mental retardation associated with iodine deficiency. The effects of excessive iodine might be subtler and, if ignored, might affect children’s life chances in ways that will go unnoticed [10], including in terms of cognitive function.

The World Health Organization/International Council for the Control of the Iodine Deficiency Disorders/United Nations Children’s Fund (WHO/ICCIDD/UNICEF) recommends median levels of urinary iodine concentration (UIC, expressed in μg of iodine per liter of urine) within the range of 100–199 µg/L for school-age children, indicating a population’s iodine adequacy [11], and the European Commission/Scientific Committee on Food (EC/SCF) has set the tolerable upper level of daily iodine intake as 250 µg/day for children between ages 4 and 6 years old, 300 µg/day between ages 7 and 10, and 450 µg/day for children aged 11 through 14 years old [9]. Iodine is present in nature in the cycle of evaporation from seawater and condensation onto land and is ingested from foods such as seafood, milk products (including breast milk), and dietary supplements, among others. A population’s median UIC, as considered by the WHO/ICCIDD/UNICEF, reflects the total iodine intake from all these sources [4]. EC/SCF’s tolerable upper intake level corresponds to the maximum amount of total chronic daily intake of iodine (from all sources) considered to be unlikely to pose risks of adverse health effects to humans.

Internationally, iodine deficiency is far more common than is excessive iodine [12,13]. For example, in 2021, only 13 countries recorded excessive iodine intakes based on the median UIC of school-age children, whereas 26 appeared as iodine-insufficient and 135 as iodine-adequate [12]. Where several studies have focused on the role of iodine deficiency on children’s cognitive function, namely in European countries [14,15], the role of iodine excess on cognition has received relatively less attention. Studies with the latter focus have been conducted in Asian countries, areas with high concentrations of iodine in drinking water [16,17,18,19,20,21]. Although there are some inconsistent results, a recent meta-analysis of such studies reported significantly lower intelligence levels among the children residing in high-iodine areas when compared to the controls [16]. However, the role of excessive iodine on child cognition among generally healthy populations from regions where the levels of iodine are less extreme remains unclear.

In Portugal, endemic goiter has disappeared since the late 1970s through the introduction of iodized salt programs in critical regions. Children’s UIC has increased over time also due to the so-called silent prophylaxis resulting from the globalization of the food chain, decreased region isolation, and the general population’s socioeconomic development [22]. For example, milk, yogurt, premade baby food, and household iodized salt were all associated with children’s iodine status in Portugal [23], and, despite the wide variation of iodine content found within the same types of food, a 2018 WHO report has indicated that a diet rich in seafood and dairy products provides the recommended daily iodine intake for a healthy adult in Portugal [24].

A previous nationwide study conducted with school children reported a median UIC of 106 μg/L. This is within the WHO’s 100–199 µg/L adequacy levels and was considered to be an improvement in iodine status compared to data from the 1980s [22]. More recently, a study from the IoGeneration project, with a sample of school-age children representative of the north of the country, reported an even greater median UIC (of 129 μg/L), with 5% of the sample registering UIC values below 50 μg/L (indicating moderate iodine deficiency) and over 5% showing excessive concentrations above 300 μg/L [23]. The goal of the current study was to inspect whether elevated iodine intake is associated with low IQ among this IoGeneration sample of school children, for whom iodine concentrations were generally adequate across all ages (the sample’s median UIC falling within the WHO’s recommended levels) but among whom a proportion already presented elevated levels.

## 2. Materials and Methods

### 2.1. Design and Participants

The IoGeneration project, of which this study is a part, assessed the iodine status and IQ of school-age children in three regions of northern Portugal (representing urban and rural, coastal and inland regions of the country) between December 2015 and May 2016 [23,25]. The methodology and population of the IoGeneration project have been described elsewhere [23]. In sum, elementary and middle school classes (1st to 6th grades) pertaining to 83 schools and 32 school clusters were selected through a multi-stage sampling procedure with three cluster levels (county, school, and school classes) for the provision of a representative sample of the population in the north of the country.

The study was conducted according to the guidelines of the Declaration of Helsinki and approved by the Ethics Committee of S. João Hospital Center/Faculty of Medicine of the University of Porto (CES 256-15, 29-09-15). Data collection was approved by the National Committee for Data Protection and by the Directorate-General of Education.

### 2.2. Measures

The IoGeneration project assessed children’s urinary iodine and creatinine excretion in one-spot, first-morning urine samples [23]. Urinary iodine is considered a good population biomarker of recent iodine intake because nearly all ingested iodine is excreted in the urine [26]. Because the ideal assessment of iodine status in several 24-h urine samples for each individual is unfeasible in large-scale studies [26], iodine status determination in random spot urine samples is considered a valuable strategy [27]. The iodine-to-creatinine ratio was used in this study because it shows a stronger correlation with 24-h urine volume than does iodine concentration [28]. Urinary iodine concentration adjusted by urinary creatinine reduces intra-individual variation in daily urine volume and in iodine excretion for same-sex groups of similar ages [29,30]. Thus, the iodine-to-creatinine ratio is more reliable than urinary iodine concentration and has been used in previous research investigating the relationship between iodine status and children’s IQs [14].

As described elsewhere [23], urinary iodine excretion was measured via inductively-coupled plasma-mass spectrometry (ICP-MS), following the method developed by the Center for Disease Control and Prevention (CDC). Creatinine was measured in an ISO 17025-accredited laboratory using an ADVIA 1800 instrument and a commercial kit (Jafe’s reaction) following the manufacturer’s instructions [31,32].

Children’s IQs were assessed with Raven’s Coloured Progressive Matrices, parallel form (CPM-P) [33], administered collectively at children’s schools by trained psychologists. Regarded as a measure of general intelligence and as a fairly pure measure of fluid reasoning [34], Raven’s Progressive Matrices have been extensively used in research [35], including in relation to children’s iodine status [7,36,37].

Information on various factors that can affect IQ was obtained through a self-report questionnaire that was designed for the IoGeneration study and sent to the parents after a pilot test [23]. Potential confounders from the questionnaire that were included in the current study were: (1) child-related aspects, namely gender, weight at birth, number of weeks of gestation, whether the child was breastfed, current intake of nutrient supplements and current consumption of seafood (fish and shellfish); (2) mother-related aspects during pregnancy or lactation, namely, intake of nutrient supplements and alcohol consumption; and indicators of the family’s living situation and socioeconomic background, namely, the mother’s age at child’s birth, number of siblings, maternal and paternal education levels, a composite measure of socioeconomic status (including house crowdedness, physical upkeep, and family’s economic hardship), and an index of the family’s atmosphere at home (i.e., levels of fighting and yelling). Similar aspects have been considered in previous research dealing with the contribution of nutrients, including iodine, to children’s cognitive development [14]. Because one of the geographical locations in this sample was an inland region that, in the past, was known for having a great incidence of thyroid disease and socio-economic disadvantages and inequalities [23], the children’s area of residence was also considered. Age was not included in the analysis because IQ percentiles were already calculated by age according to the standard procedures [38,39].

### 2.3. Analyses

Analyses were conducted in SPSS version 28. IQ scores were converted into percentiles by age and dichotomized to less than 50 (“below-average IQs”) and 50 or more [39]. A previous IoGeneration study with this sample showed that excessive iodine was especially present in the youngest age group (five-six years old) [23]; thus, children’s iodine-to-creatinine ratios were dichotomized to less than 250 µg/g and 250 µg/g or more according to the EC/SCF’s tolerable upper intake of iodine per day for this age group.

Associations between categorical variables were assessed with the *χ*^2^ test. Logistic regression was conducted to inspect the association between children’s iodine status and the odds of having an IQ below the average, with IQ percentiles on or above 50 serving as the reference group. When the full variable range was examined, analyses were based on Pearson or Spearman’s correlations and general linear model (GLM) univariate procedures.

The regression models were adjusted for various aspects that can have an impact on IQ, as mentioned above. Model 1 was adjusted for the children’s socio-demographic, economic, and living situations: gender (boy or girl), number of siblings (<2 or ≥2), mother’s age at the time of delivery, maternal and paternal education levels, a composite measure of socioeconomic status (continuous variable combining three questions, respectively, on house crowdedness, house physical upkeep, and family’s economic hardship, which all loaded onto a single factor in a component analysis conducted previously), an index of the family’s atmosphere at home (contrasting much and occasional “serious fighting and yelling” with mostly “getting along”), and region of residence (inland or coast). Model 2 was additionally adjusted for previous developmental markers and nutritional influences: birth weight (<2500 g or ≥2500 g), number of weeks of gestation (<37 weeks or ≥37 weeks), whether the child was breastfed (no or yes), mother’s intake of nutrition supplements (no or yes) and alcohol consumption (no or yes) during pregnancy/lactation, and child’s current intake of supplements (no or yes), fish (<3 times per week; ≥3 times per week), and shellfish (<1 time per month; ≥1 time per month). Statistical significance was set for *p*-values less than 0.05.

The two adjusted models included children with no missing responses in all variables, which diminished the number of participants for these two particular analyses. Comparing the group in the final model with the remainder of the IoGeneration sample, non-significant differences were found regarding iodine concentration, age, gender, and region of residence. However, children in the final model showed markers of higher socioeconomic and cultural (maternal and paternal education) status when compared with those who had missing responses and did not enter the model (supplementary material, Table S1).

## 3. Results

Of all 2018 children in the IoGeneration project, 1965 (97.4%) returned complete IQ tests and were included in the analysis after the removal of 7 cases for conditions that could affect IQ or the task of taking the IQ test (2 cases with Down syndrome, 2 with anemia and 3 with visual difficulties). The IoGeneration sample has been described elsewhere [23], and the group included in this study was equivalent to the whole IoGeneration sample regarding gender, with similar proportions of boys (*n* = 1024; 52.1%) and girls (*n* = 941; 47.9%), age distribution, ranging from 5 to 12 years old (*M* = 8.91 years old; *SD* = 1.76), and region of residence (*n* = 1152, or 58.6%, living in the coast and *n* = 813, or 41.4%, living in inland regions). Iodine status was also the same as in the whole IoGeneration sample, regarding both UIC (*Mdn* = 129 µg/L; *IQR* = 88–181 µg/L) and the iodine-to-creatinine ratio (*Mdn* = 126 µg/g; *IQR* = 83–184 µg/g). Thus, this sample’s iodine status was generally within the iodine adequacy interval of 100–199 µg/L recommended by the WHO.

Like in the whole IoGeneration sample, children’s iodine-to-creatinine ratio in this study was negatively associated with age (*r* = −0.30; *p* = 0.000) and was not associated with frequency of seafood consumption (fish: *r_s_* = 0.02; *p* = 0.482; shellfish: *r_s_* = 0.01; *p* = 0.615). It was also non-significantly associated with parental education (mother’s: *r_s_* = 0.03; *p* = 0.152; father’s: *r_s_* = 0.00; *p* = 0.872) but showed a modest positive association with socioeconomic status (*r_s_* = 0.05; *p* = 0.034).

A total of 216 children, corresponding to 11.0% of the sample in this study, had iodine-to-creatinine ratios on or above 250 µg/g, with only 15 children (0.8%) registering values above 500 µg/g. No children presented values below 20 µg/g (indicating severe iodine deficiency), and 103 (5.2%) had moderate iodine deficiency (values < 50 µg/g).

A greater proportion of children with iodine concentrations of at least 250 µg/g had below-average IQs when compared to those with less than 250 µg/g (Table 1). This was despite the presence of a sizable (though statistically non-significant) proportion of below-average IQs also at the bottom of the iodine distribution for moderate iodine deficiency (53/103 = 51.5% in the <50 µg/g group *versus* 916/1862 = 49.2% in the ≥50 µg/g group, *p* = 0.655). Subdividing the high-iodine group, the proportion of children with below-average IQs increased from the <250 µg/g group to the 250–399 µg/g group and to the ≥400 µg/g group (Table 1).

Table 2 shows the results of the logistic regression. The association of iodine status with IQ remained significant even after the adjustment for potential confounders, and the odds ratios (ORs) did not show important changes from the unadjusted to the two adjusted models (OR 1.35, 1.46, and 1.55, respectively). Children with iodine concentrations of at least 250 µg/g were more likely to have below-average IQs than children with concentrations of less than 250 µg/g.

Similar results were obtained when the high-iodine group was subdivided (despite the loss of power), adjusting for confounders. Table 3 shows the same logistic regression for the subdivided elevated iodine category as before (model 2), additionally considering the group with iodine concentrations below 50 µg/g for comparison. The category of reference was the 50–249 µg/g group. Again, the odds of having below-average IQs increased for increasingly high iodine status (*p* = 0.037), with the group on or above 400 µg/g appearing as particularly critical.

When the full range of IQ values was considered, the results of the GLM univariate analysis were equivalent. Adjusting for the effect of confounders (model 2), children in the iodine group on or above 250 µg/g displayed a lower mean IQ (*M* = 46.22; *SD* = 27.89) than children in the less-than-250 µg/g group (*M* = 52.52; *SD* = 28.49), *F*(1) = 7.501; *p* = 0.006.

In sensitivity analyses, testing whether the associations between iodine status and IQ might possibly be confounded by the children’s urinary concentrations of lead and of other heavy metals (from foods or other possible sources) added to model 2 (aluminum, manganese, cobalt, nickel, copper, arsenic, molybdenum, cadmium, tin, antimony, thallium), all adjusted for urinary creatinine, did not alter the results (supplementary material, Table S2a,b). A subgroup analysis of 616 children who participated in an additional on-line questionnaire about the frequency of consumption of dairy products (e.g., milk and eggs), where these possible confounders were added to model 2, also yielded similar results (supplementary material, Table S3). The iodine-to-creatinine ratio was additionally tested as a continuous variable and the data remained unchanged as well (supplementary material, Table S4). Testing whether possible interactions between iodine intake (<250 µg/g or ≥250 µg/g) and the confounders might be influencing the results (with IQ percentiles as the dependent variable) also yielded null results (supplementary material, Table S5).

## 4. Discussion

Research on the effects of excessive iodine on the IQ of children have been conducted in Asian regions where iodine concentrations in drinking water are very high [16]. Despite some inconsistent results, in general, these studies point to the presence of decreased intelligence levels among children residing in high-iodine areas, namely, when compared with their lower-iodine counterparts [16,17,18]. The current study sought to investigate the association between elevated iodine intake and the IQ of children among generally healthy populations from regions with less extreme endemic levels of iodine, where research is lacking, and iodine supplementation policies might exist.

A previous IoGeneration study showed that a representative sample of children from northern Portugal presented an adequate median iodine status, following WHO’s 100–199 µg/L UIC recommendations [23]. The results in the current study indicate that, in that IoGeneration sample, the children presenting high iodine levels (≥250 µg/g) had increased odds of having below-average IQs, when compared to those in the <250 µg/g group. The results in this Western sample of children are thus in line with the research in elevated-iodine Asian regions regarding the association between high-iodine intake and IQ [16,17,18].

Iodine is ingested from various sources, and a population’s iodine status reflects the total iodine intake from all these sources [4]. Milk is an important source of iodine in several countries [4,24], and the negative correlation found between children’s age and iodine status might have to do with milk consumption decreasing as children grow older, as has been proposed previously [23,40]. Despite WHO’s indication that a diet rich in seafood can contribute to the recommended daily iodine intake for a healthy adult in Portugal [24], seafood consumption was non-significantly correlated with children’s iodine levels. This might be due to the types of seafood that children tend to consume because there is a wide variation of iodine content in different kinds of seafood [24]. Children in Portugal seem to be ingesting iodine from other sources, namely milk and yogurt [23]. Although parental education was uncorrelated with children’s iodine levels, a modest correlation was found between socioeconomic status and iodine intake in this study. This finding suggests that, at a population level, the greater the socioeconomic status, the greater the amount of iodine intake, which could pose a challenge regarding problematic elevated iodine intake occurring especially among higher socioeconomic levels. Nevertheless, when the models were adjusted for other risk factors (discussed below), socioeconomic status was among the aspects that showed non-significant associations with IQ.

The important adverse effects of iodine deficiency on child IQ have been documented in European countries [14,15]. This study adds the negative effects of high iodine intake on child IQ to the existing research. The data show an increasing trend in the proportion of below-average IQs as children’s high-iodine concentrations increased. The fact that the association between iodine status and IQ remained significant even after adjusting for various potential confounders reinforces the presence of a negative effect of elevated iodine intake on children’s cognitive function.

Other risk factors significantly associated with below-average IQs in the models included the area of residence, supporting the possibility of social inequalities existing between the country’s inland and coastal regions [23]. All else being equal, the decreasing age of the mother at childbirth was also associated with the risk of the offspring having below-average IQs, in line with past research [41]. Parents’ education has been one of the aspects that have shown the strongest associations with children’s cognitive development [42] and was significantly related to child IQ in this study as well. Seafood consumption was uncorrelated with iodine status in this study, so the association found between less-frequent consumption of fish and below-average IQ might have to do with other important nutrients that fish provide, including omega-3 long-chain polyunsaturated fatty acids and others, and is in line with previous research also suggesting a positive association between fish consumption and children’s fluid intelligence [43]. Various factors can affect children’s IQs, and past research associating nutrients, including iodine, with children’s intelligence, has controlled analyses for these potential confounders to provide increasingly adjusted models of that association [6,14,43]. Nevertheless, the remaining risk factors included in this study showed non-significant relations with children’s IQs in the models.

This study dealt with children’s fluid intelligence. Future studies are necessary to examine other cognitive outcomes as well. As subtle as the effects of excessive iodine on cognitive functioning might be, they can potentially affect children’s life chances, namely in terms of academic and eventual economic success, among other outcomes. This study had some other limitations. Because of its observational design, residual confounding variables might have been left out, although the adjustment for a large number of potential confounders in the study yielded little changes in the size and significance of the effects. Second, the assessment of a one-spot urine sample per individual has limitations [29,30], and the assessment of iodine status restricted to one point in time might not represent the child’s actual dietary iodine intake or thyroid function. Efforts to minimize these limitations in this study included the correction for urinary volume through the use of the iodine-to-creatinine ratio (though this adjustment also has limitations [27]) and the classification of the children into two broad groups of iodine status, thereby reducing the possibility of misclassification of that status. Third, only children with no missing values in all the variables were included in the two adjusted regression models, and the results for that particular analysis might be somewhat different if the entire sample had been included. Otherwise, the group in this study consisted practically of the whole IoGeneration sample.

The results of the current study indicate that, even in an iodine-adequate sample, high iodine levels can increase the odds of children having below-average IQs. These findings need to be replicated with other samples, particularly in regions where iodine concentrations are not as extreme as in those regions where research on excessive iodine tends to occur. The present results, in terms of cognitive function, bear on controlled trials and on policies involving iodine supplementation, especially among generally healthy populations already presenting adequate levels of iodine.

## Figures and Tables

**Table 1:** Proportion of school children (ages 6 through 12 years) presenting below-average IQs by iodine status (*N* = 1965).

	Iodine Status			Subdivided High Iodine Status			
	<250 µg/g	≥250 µg/g	*p* ^1^	<250 µg/g	250–399 µg/g	≥400 µg/g	*p* ^1^
Below-average IQ	848/1749 (48%)	121/216 (56%)	0.037	848/1749 (48%)	96/178 (54%)	25/38 (66%)	0.047

Iodine status = urinary iodine-to-creatinine ratio (µg/g). Below-average IQ = intelligence quotient percentiles < 50 (vs. ≥50). ^1^ *χ*^2^ test.

**Table 2:** Odds of lower IQ for high urinary iodine-to-creatinine ratio, unadjusted and adjusted for possible confounders.

	Below-Average IQ					
	Unadjusted Model (*N* = 1965)		Adjusted Model 1 (*N* = 1814)		Adjusted Model 2 (*N* = 1552)	
	OR (95% CI)	*p*	OR (95% CI)	*p*	OR (95% CI)	*p*
Iodine status ≥ 250 µg/g	1.35 (1.02–1.80)	0.037	1.46 (1.07–1.98)	0.016	1.55 (1.11–2.17)	0.011
Male gender			1.14 (0.94–1.38)	0.187	1.22 (0.99–1.50)	0.065
Number of siblings ≥ 2			1.21 (0.94–1.55)	0.143	1.22 (0.92–1.61)	0.170
Mother’s younger age at delivery			1.03 (1.02–1.05)	0.000	1.03(1.01–1.05)	0.006
Mother’s lower education level			1.09 (1.05–1.13)	0.000	1.08 (1.03–1.12)	0.001
Father’s lower education level			1.07 (1.03–1.11)	0.000	1.07 (1.03–1.12)	0.001
Lower socioeconomic score			1.02 (0.81–1.29)	0.857	1.01 (0.78–1.31)	0.946
Difficult atmosphere at home			0.95 (0.75–1.21)	0.664	0.92 (0.70–1.20)	0.523
Living away from the Coast			1.34 (1.10–1.64)	0.004	1.31 (1.05–1.63)	0.016
Birth weight < 2500 g					0.89 (0.58–1.36)	0.591
Weeks of gestation < 37					1.02 (0.71–1.48)	0.906
Child not breastfed					1.06 (0.78–1.42)	0.720
Mother not using supplements in pregnancy/breastfeeding					1.01 (0.79–1.29)	0.950
Mother using alcohol in pregnancy/breastfeeding					0.79 (0.55–1.14)	0.203
Child not using supplements currently					0.80 (0.51–1.25)	0.332
Fish intake < 3 times per week					1.37 (1.10–1.71)	0.006
Shellfish intake < 1 time per month					0.89 (0.63–1.25)	0.490

Below-average IQ = intelligence quotient percentiles < 50 (vs. ≥ 50). OR = odds ratio. CI = confidence interval.

**Table 3:** Odds of lower IQ for subdivided elevated urinary iodine concentrations (also considering values below 50 µg/g), adjusted for possible confounders.

			Below-Average IQ	
			Adjusted Model 2 (*N* = 1552)	
			OR (95% CI)	*p*
Iodine status				0.037
	<50 µg/g ^a^	*n* = 85	1.15 (0.73–1.82)	0.557
	250–399 µg/g ^a^	*n* = 141	1.42 (0.98–2.04)	0.061
	≥400 µg/g ^a^	*n* = 29	2.61 (1.15–5.95)	0.022
Male gender			1.22 (0.99–1.51)	0.063
Number of siblings ≥ 2			1.21 (0.91–1.60)	0.186
Mother’s younger age at delivery			1.03 (1.01–1.05)	0.006
Mother’s lower education level			1.08 (1.03–1.12)	0.001
Father’s lower education level			1.07 (1.03–1.12)	0.001
Lower socioeconomic score			1.01 (0.78–1.31)	0.937
Difficult atmosphere at home			0.92 (0.70–1.19)	0.519
Living away from the Coast			1.32 (1.06–1.64)	0.014
Birth weight < 2500 g			0.88 (0.58–1.35)	0.559
Weeks of gestation < 37			1.02 (0.71–1.48)	0.900
Child not breastfed			1.05 (0.78–1.41)	0.757
Mother not using supplements in pregnancy/breastfeeding			1.01 (0.78–1.29)	0.958
Mother using alcohol in pregnancy/breastfeeding			0.79 (0.55–1.14)	0.207
Child not using supplements currently			0.81 (0.52–1.26)	0.345
Fish intake < 3 times per week			1.36 (1.09–1.70)	0.007
Shellfish intake < 1 time per month			0.88 (0.62–1.24)	0.463

Below-average IQ = intelligence quotient percentiles < 50 (vs. ≥ 50). OR = odds ratio. CI = confidence interval. ^a^ Comparing with the category of reference: 50–249 µg/g.

## Data Availability

The study protocol can be found at https://clinicaltrials.gov/ct2/show/NCT02608138, accessed on 9 September 2022. Data described in the manuscript will be made available upon request pending application and approval.

## Supplementary Materials

The following supporting information can be downloaded at: https://www.mdpi.com/article/10.3390/nu14214493/s1, Table S1. Differences between participants in the final regression model and in the whole IoGeneration sample; Table S2a. Odds of lower IQ for elevated urinary iodine-to-creatinine ratio adjusted for lead and other possible confounders; Table S2b. Odds of lower IQ for elevated urinary iodine-to-creatinine ratio adjusted for heavy metals and other possible confounders; Table S3. Odds of lower IQ for elevated urinary iodine-to-creatinine ratio adjusted for frequency of consumption of dairy products (milk and eggs) and other possible confounders (subgroup analysis); Table S4. Odds of lower IQ for urinary iodine-to-creatinine ratio treated as a continuous variable, adjusted for possible confounders; Table S5. Effect of interactions between iodine intake (<250 µg/g or ≥250 µg/g) and confounders on children’s IQ percentiles.
