# Supplementary material for: Association between Elevated Iodine Intake and IQ among School Children in Portugal
Source: Nutrients. 2022 Oct 26;14(21):4493. doi: 10.3390/nu14214493 (PMC9657725; doi:10.3390/nu14214493)
Supplement: Supplementary file 1 [file nutrients-14-04493-s001.zip › nutrients-1936997-supplementary.pdf]

Supplementary material

Table of contents

Table S1:.Differences between participants in the whole IoGeneration sample and in the final regression model p. 2

Table S2a. Odds of lower IQ for elevated urinary iodine-to-creatinine ratio adjusted for lead and other possible confounders p. 3

Table S2b. Odds of lower IQ for elevated urinary iodine-to-creatinine ratio adjusted for heavy metals and other possible confounders p. 4

Table S3. Odds of lower IQ for elevated urinary iodine-to-creatinine ratio adjusted for frequency of consumption of dairy products (milk and eggs) and other possible confounders (subgroup analysis) p. 5

Table S4. Odds of lower IQ for urinary iodine-to-creatinine ratio treated as a continuous variable, adjusted for possible confounders p. 6

Table S5. Effect of interactions between iodine intake (< 250 µg/g or ≥ 250 µg/g) and confounders on children's IQ percentiles p. 7

Table S1. Differences between participants in the final regression model and in the whole IoGeneration sample

		N			p^1
Gender - *n (%)*					
	Total sample	2018	Boys: 1050 (52%)	Girls: 968 (48%)	0.494
	In the model	1552	Boys: 814 (52%)	Girls: 738 (48%)	
Region of residence - *n (%)*					
	Total sample	2018	Coast: 1187 (59%)	Inland: 831 (41%)	0.839
	In the model	1552	Coast: 911 (59%)	Inland: 641 (41%)	
Age - *M*; *SD*					
	Total sample	2018	8.90	1.75	0.918
	In the model	1552	8.91	1.75	
Urinary iodine concentration (µg/L) - *mdn*; *P25-P75*					
	Total sample	2013	129	88-181	0.458
	In the model	1552	129	88-181	
Iodine-to-creatinine ratio (µg/g) - *mdn*; *P25-P75*					
	Total sample	2013	126	83-184	0.358
	In the model	1552	126	83-184	
Socioeconomic status - *M*; *SD*					
	Total sample	1967	3.03	0.45	0.000
	In the model	1552	3.07	0.42	
Mother's education level - *M*; *SD*					
	Total sample	1959	9.35	3.23	0.000
	In the model	1552	9.61	3.11	
Father's education level - *M*; *SD*					
	Total sample	1921	8.50	3.18	0.000
	In the model	1552	8.65	3.14	

[1] Differences were inspected with *t*-tests or $\chi 2$ tests; *M* = mean; *SD* = standard deviation; *mdn* = median; *P*25 = percentile 25; *P*75 = percentile 75.

Table S2a. Odds of lower IQ for elevated urinary iodine-to-creatinine ratio adjusted for lead and other possible confounders

	Below-average IQ	
	Adjusted model 2 (*N* = 1516)	
	OR (95% CI)	p^1
Iodine status (≥ 250μg/g)	1.59 (1.13-2.24)	0.008
Male gender	1.21 (0.98-1.50)	0.080
Number of siblings ≥ 2	1.23 (0.93-1.64)	0.146
Mother's younger age at delivery	1.03 (1.00-1.05)	0.019
Mother's lower education level	1.08 (1.04-1.13)	0.000
Father's lower education level	1.07 (1.03-1.12)	0.001
Lower socioeconomic score	1.01 (0.77-1.32)	0.949
Difficult atmosphere at home	0.91 (0.70-1.20)	0.513
Living away from the Coast	1.35 (1.08-1.69)	0.008
Birth weight < 2500 g	0.90 (0.58-1.38)	0.615
Weeks of gestation < 37	1.01 (0.70-1.47)	0.948
Child not breastfed	1.04 (0.77-1.41)	0.799
Mother not using supplements in pregnancy/breastfeeding	1.05 (0.81-1.35)	0.724
Mother using alcohol in pregnancy/breastfeeding	0.80 (0.55-1.15)	0.226
Child not using supplements currently	0.86 (0.54-1.36)	0.513
Fish intake < 3 times per week	1.40 (1.11-1.75)	0.004
Shellfish intake < 1 time per month	0.91 (0.63-1.29)	0.581
Lead per creatinine in urine (μg/g)	0.97 (0.86-1.09)	0.612

Below-average IQ = intelligence quotient percentiles < 50 (vs. ≥ 50). OR = odds ratio. CI = confidence interval. [1] From binary logistic regression.

Table S2b. Odds of lower IQ for elevated urinary iodine-to-creatinine ratio adjusted for heavy metals and other possible confounders

	Below-average IQ	
	Adjusted model 2 ($N = 1236$)	
	OR (95% CI)	p[1]
Iodine status (≥ 250µg/g)	1.56 (1.06-2.30)	0.025
Male gender	1.31 (1.03-1.66)	0.028
Number of siblings ≥ 2	1.34 (0.97-1.84)	0.073
Mother's younger age at delivery	1.03 (1.01-1.05)	0.015
Mother's lower education level	1.08 (1.03-1.14)	0.001
Father's lower education level	1.07 (1.02-1.12)	0.008
Lower socioeconomic score	1.00 (0.73-1.34)	0.936
Difficult atmosphere at home	0.99 (0.73-1.35)	0.968
Living away from the Coast	1.46 (1.11-1.93)	0.007
Birth weight < 2500 g	0.97 (0.60-1.57)	0.914
Weeks of gestation < 37	1.06 (0.70-1.60)	0.779
Child not breastfed	1.05 (0.75-1.46)	0.796
Mother not using supplements in pregnancy/breastfeeding	1.03 (0.77-1.37)	0.853
Mother using alcohol in pregnancy/breastfeeding	0.78 (0.52-1.17)	0.232
Child not using supplements currently	0.83 (0.49-1.42)	0.499
Fish intake < 3 times per week	1.38 (1.07-1.78)	0.013
Shellfish intake < 1 time per month	0.93 (0.63-1.37)	0.705
Lead per creatinine in urine (µg/g)	0.92 (0.78-1.08)	0.301
Aluminum per creatinine in urine (µg/g)	1.01 (0.99-1.02)	0.116
Manganese per creatinine in urine (µg/g)	1.03 (0.94-1.14)	0.485
Cobalt per creatinine in urine (µg/g)	0.97 (0.78-1.21)	0.809
Nickel per creatinine in urine (µg/g)	1.00 (0.98-1.02)	0.888
Copper per creatinine in urine (µg/g)	1.00 (0.99-1.01)	0.857
Arsenic per creatinine in urine (µg/g)	1.00 (0.99-1.00)	0.600
Molybdenum per creatinine in urine (µg/g)	1.00 (0.99-1.00)	0.726
Cadmium per creatinine in urine (µg/g)	0.72 (0.21-2.48)	0.604
Tin per creatinine in urine (µg/g)	1.04 (0.99-1.10)	0.100
Antimony per creatinine in urine (µg/g)	0.90 (0.62-1.32)	0.593
Thallium per creatinine in urine (µg/g)	0.68 (0.49-0.95)	0.022

Below-average IQ = intelligence quotient percentiles < 50 (vs. ≥ 50). OR = odds ratio. CI = confidence interval. [1] From binary logistic regression.

Table S3. Odds of lower IQ for elevated urinary iodine-to-creatinine ratio adjusted for frequency of consumption of dairy products (milk and eggs) and other possible confounders (subgroup analysis)

	Below-average IQ	
	Adjusted model 2 (N = 512)	
	OR (95% CI)	p^1
Iodine status (≥ 250µg/g)	2.36 (1.29-4.32)	0.005
Male gender	1.15 (0.79-1.69)	0.468
Number of siblings ≥ 2	1.16 (0.67-2.00)	0.594
Mother's younger age at delivery	1.02 (0.98-1.07)	0.263
Mother's lower education level	1.18 (1.08-1.30)	0.000
Father's lower education level	1.02 (0.94-1.11)	0.596
Lower socioeconomic score	0.65 (0.38-1.11)	0.113
Difficult atmosphere at home	1.18 (0.71-1.96)	0.519
Living away from the Coast	1.57 (1.03-2.40)	0.038
Birth weight < 2500 g	0.70 (0.29-1.66)	0.415
Weeks of gestation < 37	1.18 (0.61-2.30)	0.626
Child not breastfed	0.74 (0.38-1.44)	0.374
Mother not using supplements in pregnancy/breastfeeding	0.67 (0.37-1.22)	0.191
Mother using alcohol in pregnancy/breastfeeding	0.68 (0.34-1.34)	0.265
Child not using supplements currently	0.69 (0.35-1.39)	0.305
Fish intake < 3 times per week	1.31 (0.88-1.97)	0.189
Shellfish intake < 1 time per month	1.48 (0.69-3.17)	0.318
Milk intake	1.43 (0.91-1.26)	0.123
Eggs intake	0.91 (0.59-1.40)	0.667

Below-average IQ = intelligence quotient percentiles < 50 (vs. ≥ 50). OR = odds ratio. CI = confidence interval. [1] From binary logistic regression.

Table S4. Odds of lower IQ for urinary iodine-to-creatinine ratio treated as a continuous variable, adjusted for possible confounders

	Below-average IQ	
	Adjusted model 2 ($N = 1552$)	
	**OR (95% CI)**	p^1
Iodine status (continuous variable)[a]	1.01 (1.00-1.02)	0.033
Male gender	1.21 (0.98-1.50)	0.070
Number of siblings ≥ 2	1.21 (0.92-1.61)	0.174
Mother’s younger age at delivery	1.03 (1.01-1.05)	0.005
Mother’s lower education level	1.08 (1.03-1.12)	0.000
Father’s lower education level	1.07 (1.03-1.12)	0.001
Lower socioeconomic score	1.00 (0.77-1.30)	0.987
Difficult atmosphere at home	0.92 (0.71-1.20)	0.540
Living away from the Coast	1.30 (1.05-1.62)	0.019
Birth weight < 2500 g	0.88 (0.58-1.35)	0.554
Weeks of gestation < 37	1.05 (0.73-1.52)	0.801
Child not breastfed	1.05 (0.78-1.42)	0.740
Mother not using supplements in pregnancy/breastfeeding	1.01 (0.79-1.30)	0.931
Mother using alcohol in pregnancy/breastfeeding	0.79 (0.55-1.13)	0.203
Child not using supplements currently	0.77 (0.50-1.20)	0.253
Fish intake < 3 times per week	1.38 (1.10-1.72)	0.005
Shellfish intake < 1 time per month	0.89 (0.63-1.25)	0.490

Below-average IQ = intelligence quotient percentiles < 50 (vs. ≥ 50). OR = odds ratio. CI = confidence interval. [a] Iodine-to-creatinine ratio expressed as tens of µg/g (i.e., each additional ten µg/g of iodine results in a 1% increase in the odds of a below-average IQ). [1] From binary logistic regression.

Table S5. Effect of interactions between iodine intake (< 250 µg/g or ≥ 250 µg/g) and confounders on children's IQ percentiles

	IQ percentiles		
	Adjusted model 2 (*N* = 1552)		
	F	***Degrees of freedom***	***p*[1]**
Gender * Iodine status	0.662	1	0.416
No. of siblings * Iodine status	0.926	1	0.336
Mother's age at delivery * Iodine status	0.784	25	0.767
Mother's education * Iodine status	1.024	9	0.418
Father's education * Iodine status	1.361	9	0.201
Socio-economic level * Iodine status	0.966	6	0.447
Atmosphere at home * Iodine status	0.124	1	0.725
Region of residence * Iodine status	0.000	1	0.985
Birth weight * Iodine status	1.555	1	0.213
Weeks of gestation * Iodine status	0.509	1	0.475
Breastfeeding * Iodine status	0.358	1	0.549
Mother's supplement use during pregnancy/breastfeeding * Iodine status	0.304	1	0.582
Mother's alcohol use during pregnancy/breastfeeding * Iodine status	0.218	1	0.640
Child's current supplement use * Iodine status	1.281	1	0.258
Fish intake * Iodine status	0.352	1	0.553
Shellfish intake * Iodine status	0.044	1	0.834

[1] From GLM univariate analysis.
